# Silicon-coated carbon quantum dots composite nanomaterials mediate pest resistance activation in tobacco (*Nicotiana tabacum*)

**DOI:** 10.1186/s12951-025-03449-0

**Published:** 2025-05-19

**Authors:** Kang-Li He, Jing-Han Yang, Fu-Xiao Yu, Nuo Wei, Qian-Wei Liang, Jia-Wei Feng, Tian-Ci Yi, Xiang-Sheng Chen, Guy Smagghe, Shun-Hua Gui, Tong-Xian Liu

**Affiliations:** 1 Guizhou Provincial Key Laboratory for Agricultural Pest Management of the Mountainous Region, Institute of Entomology, College of Agriculture, Guizhou University, Guiyang, 550025 China; 2 Institute of Plant Health and Medicine, Guizhou University, Guiyang, 550025 China; 3 State Key Laboratory of Green Pesticide, Key Laboratory of Green Pesticide and Agricultural Bioengineering, Ministry of Education, Center for Research and Development of Fine Chemicals of Guizhou University, Guiyang, 550025 China; 4 College of Plant Protection, Yangzhou University, Yangzhou, 225009 China

**Keywords:** Nanomaterials, Tobacco, Plant resistance, Crop protection, Biosafety

## Abstract

**Background:**

Plant resistance inducers based on nanomaterials (NMs) are a cutting-edge and promising field of interdisciplinary research, focused on developing environmentally and ecologically friendly alternatives for protecting crops. Studies have shown that NMs composed of silicon (SiO_2_) and carbon quantum dots (CDs) can help plants better withstand various environmental and pest-related stresses.

**Results:**

We synthesized and characterized SiO_2_-coated CDs (SiO_2_@CDs) NMs that were found to be absorbed by tobacco leaves. Our research demonstrated that spraying tobacco leaves with a solution containing 100 mg/L SiO_2_@CDs was more effective in promoting plant growth and controlling pest populations, specifically adult aphids compared to using either CDs or SiO_2_ alone at the same concentration. The group treated with SiO_2_@CDs achieved a significant 71% mortality of adult aphids after just 7 days, which was significantly different from the control group. Mechanistically, SiO_2_@CDs enhanced both the plant’s physical resistance by utilizing Si, and stimulated the production of chemical defense compounds (such as salicylic acid), thereby improving aphid resistance. Additionally, the application of SiO_2_@CDs significantly reduced oxidative stress in the leaves caused by aphid infestation, bolstered the activity of antioxidant enzymes like superoxide dismutase and peroxidase, and reduced malondialdehyde accumulation. Our biosafety experiments indicated that the SiO_2_@CDs were less toxic and safer for non-target organisms in the environment, as well as for human cells.

**Conclusion:**

This study demonstrates that SiO_2_@CDs exhibit excellent performance as a multifunctional insecticide in managing aphid-induced plant pest infestations, highlighting their promising and environmentally friendly potential in pest control and agroecosystem optimization.

**Supplementary Information:**

The online version contains supplementary material available at 10.1186/s12951-025-03449-0.

## Graphical Abstract

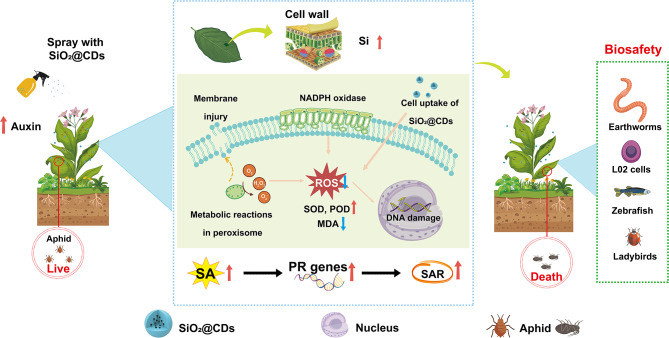

## Introduction

Tobacco (*Nicotiana tabacum*), a member of the Solanaceae family, is a highly valued economic crop with global significance. Its primary use in cigarette production is well-known, but it also holds immense potential in the medical field. Research has shown that tobacco can be utilized in vaccine development and the creation of therapeutic proteins, underscoring its important role in society [1, 2]. However, one major challenge faced by tobacco growers is the frequent pest infestation, particularly aphids and whiteflies. These pests can significantly reduce the crop’s yield and quality, posing a serious threat to the cultivation of tobacco [3–5].

In response to this challenge, numerous studies have shown that strengthening the self-defense mechanisms of tobacco to combat pests can be an effective strategy [6–8]. This approach can trigger the plant’s endogenous immune responses, significantly improving the crop’s ability to fend off pests and diseases [9, 10]. Specifically, when plants are attacked by pests, they activate signaling pathways and antioxidant systems, reducing oxidative stress and maintaining cellular balance, thus boosting their resistance to pests [11–13]. Generally, there are two main ways to enhance plant resistance: genetically modified (GM) crops and induced resistance. GM crops involve introducing disease-resistance genes or increasing the expression of endogenous resistance genes to enhance defense against specific diseases. On the other hand, induced resistance is a plant’s defensive response to external stimuli like plant pathogens, mechanical damage, or certain exogenous compounds [14–16]. Compared to the GM crops, induced resistance is more versatile and can activate a variety of plant defense mechanisms, making it applicable to a wide range of crops with different types of pests and diseases [17, 18]. Induced resistance has been gaining attention as an effective and practical strategy for enhancing plant defense in recent years due to its flexibility and adaptability to different pests and diseases.

Traditional methods of inducing resistance typically involve the use of chemical or biological inducers [19–21]. While effective in activating the plant’s immune response, these methods often have limitations in practical applications. Inducers can be unstable within plants, have a short duration of effect, and are easily influenced by external environmental conditions. These challenges hinder the widespread use and long-term efficacy of induced resistance strategies [22, 23]. Fortunately, advancements in material sciences have introduced nanomaterials (NMs) as a promising tool for inducing plant resistance [24, 25]. Carbon quantum dots (CDs), in particular, have shown several unique advantages in enhancing plant resistance. CDs have excellent optical properties that allow them to effectively absorb and convert light energy, thereby improving the efficiency of photosynthesis. This is particularly beneficial during environmental stress when plants experience disruptions in photosynthesis. CDs can help plants utilize light energy effectively, supporting normal photosynthetic activity, thus promoting growth, and enhancing their tolerance to adverse environmental conditions [26–28]. Additionally, CDs can boost the activity of antioxidant enzymes, such as peroxidase (POD) and catalase (CAT), which play a crucial role in assisting plants to eliminate excess reactive oxygen species (ROS) during pathogen attacks or environmental stress [29]. This helps maintain redox homeostasis within cells. Zhong et al. (2023) prepared spermine-CDs that have been shown to significantly increase heat resistance and activate POD and CAT activities in tomato plants by regulating photosynthesis and cellular redox homeostasis [30].

While CDs have demonstrated numerous advantages in enhancing plant resistance, they also come with certain limitations. One of the main drawbacks is their susceptibility to degradation under high temperatures and intense light, leading to the production of toxic by-products that can pose risks to the environment and human health [31–34]. These challenges have limited their practical application of CDs in the field, highlighting the need to improve their stability and prevent the formation of harmful compounds. To address this issue, researchers have turned to material modifications, with a focus on utilizing silicon (SiO_2_) nanoparticles due to their excellent chemical stability and biocompatibility. By encapsulating CDs with SiO_2_, an additional layer of protection can be provided, safeguarding the CDs from degradation in extreme conditions and further enhancing their potential use in plants [35–37]. Additionally, the SiO_2_ can accumulate in plant cell walls, forming a silica layer that increases the hardness and thickness of the cell walls. This, in turn, helps plants to resist physical damage caused by pests and diseases, as well as environmental stressors [38–40]. In essence, the strategy of combining CDs with SiO_2_ offers a comprehensive solution to overcome the limitations of CDs and effectively enhance plant resistance. While there is a growing interest in using SiO_2_-encapsulated CDs to boost plant resistance, there is still a lack of research in this area. Additionally, the specific mechanisms through which this composite material enhances resistance in tobacco plants remain to be explored.

**Scheme 1:**
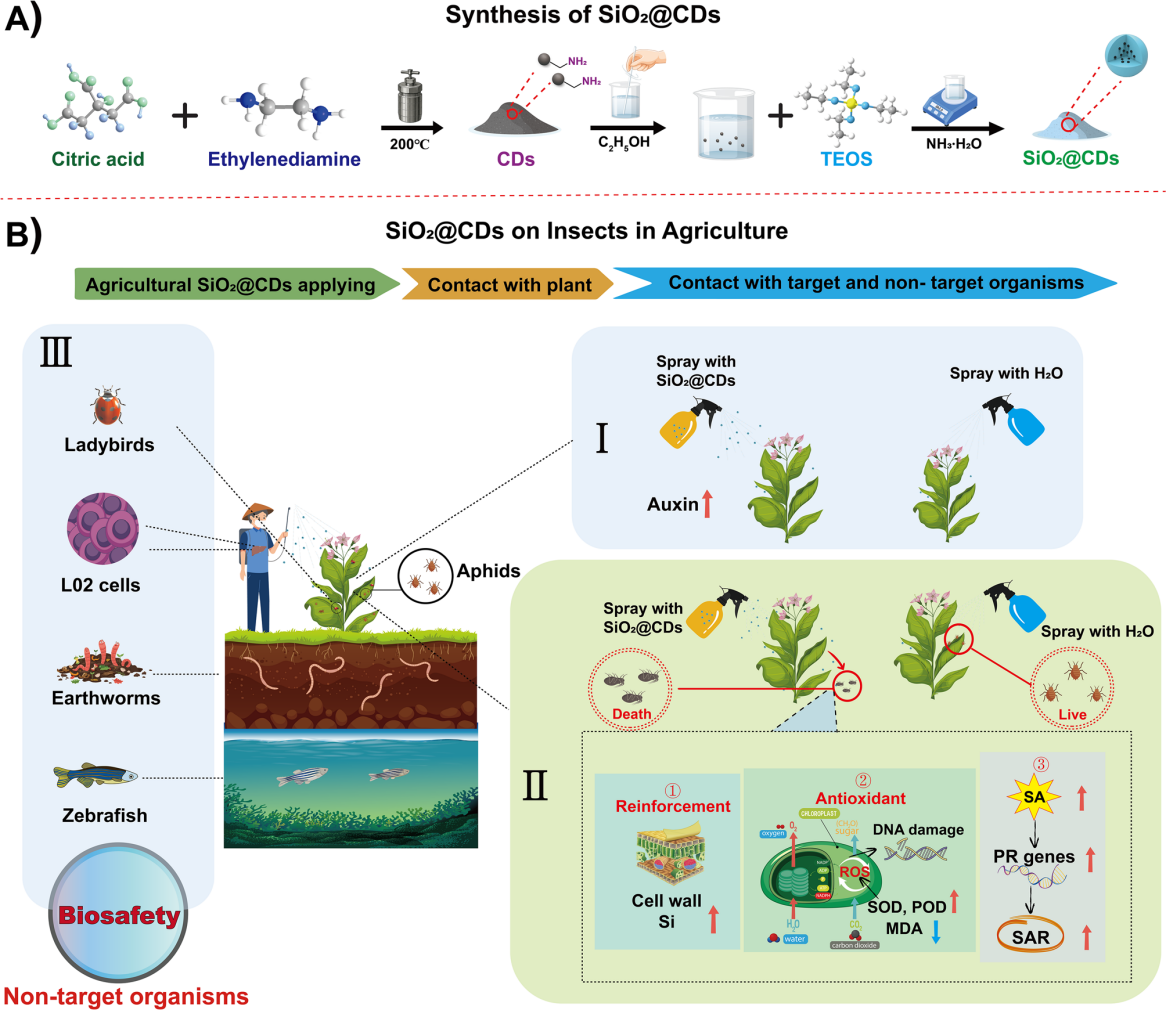
Schematic depiction of constructing composite nanomaterials (SiO_2_@CDs) to promote plant growth and enhance resistance to pests, while also ensuring safety for non-target organisms

This study utilized a simple sol-gel method to synthesize a SiO_2_-coated CDs composite (SiO_2_@CDs) (as illustrated in Scheme 1A), with the goal of controlling tobacco aphids (*Myzus persicae*), an important pest of tobacco plants [41, 42]. The study delved into the following key issues: (1) assessing the efficacy of different materials (CDs, SiO_2_ and SiO_2_@CDs) in promoting tobacco plant growth and controlling pest aphids; (2) elucidating the specific mechanisms through which SiO_2_@CDs bolster tobacco plant resistance to aphids; and (3) evaluating the environmental safety of SiO_2_@CDs in practical application (Scheme 1B). The objective of this study is to elucidate the potential of nanocomposites (SiO_2_@CDs) in enhancing tobacco plant’s resistance and ensuring biosafety. This study aims to establish a scientific foundation and provide guidance for the use of exogenous NMs in fostering plant resistance. 

## Materials and methods

### Materials and instruments

Ethylenediamine (98%, RG, Adamas), citric acid (≥ 99.5%, AR, Greagent), anhydrous ethanol (C_2_H_5_OH, ≥ 99.7%, AR), ammonia (NH_3_‧H_2_O, 25–28%, AR), tetraethyl orthosilicate (TEOS, 98%, AR), were purchased from Shanghai Titan Scientific (Xuhui, Shanghai, China). The CCK-8 kit was purchased from Invigentech (IV08-100) while the live/dead cell staining kit was acquired from BestBio (Pudong, Shanghai, China). All chemicals were used directly as obtained, without undergoing additional purification.

The tobacco aphids (*M. persicae*) used in this study were originally collected from tobacco fields located in the Huaxi District of Guizhou Province, China, and have been maintained under standardized conditions in the Insect Research Institute of Guizhou University for five years. Tobacco plants (cultivar K326, donated by Fenggang Biological Control Predator Breeding Center, Zhunyi, Guizhou) were cultivated in seedling trays within a controlled walk-in environment chamber at the same institute mirroring the conditions of the aphids. Earthworms (*Eisenia fetida*), blue zebrafish (*Danio rerio*), and ladybirds (*Hippodamia variegate*) were obtained from Guizhou Zhuoyi Biotechnology (Guiyang, Guizhou, China) for subsequent experiments under standardized conditions. L02 cells were purchased from iCell Bioscience (Fengxian, Shanghai, China) for cellular experiments.

The morphology of SiO_2_ and its SiO_2_@CDs complex was analyzed using Transmission Electron Microscopy (TEM) (Tecnai G2, FEI, Hillsboro, USA). High-resolution TEM (HRTEM) with a JEM-F200 microscope (JEOL, Japan) was used to study the morphology of CDs. A Scanning Transmission Electron Microscopy (SEM) (S-4800, Hitachi, Japan) was used to investigate the microstructures of CDs, SiO_2_, and SiO_2_@CDs samples. A Fourier-transform infrared (FT-IR) spectrophotometer (Antains II, Thermo Fisher Scientific, Waltham, MA, USA) was used for the analysis of the functional groups present in the CDs, SiO_2_, and SiO_2_@CDs composite material. Ultraviolet-visible (UV-vis) spectra were measured using a UV–2335 spectrophotometer (Uniko, Shanghai, China). The X-ray diffraction (XRD) spectra of CDs, SiO_2_ and SiO_2_@CDs were obtained using a Smart Lab diffractometer (Rigaku D/max-A, Tokyo, Japan). The X-ray photoelectron spectroscopy (XPS) of CDs, SiO_2_, and SiO_2_@CDs was performed using K-Alpha (Thermo Fisher Scientific, Waltham, MA, USA). Fluorescence spectra and photostability were recorded on a Fluoromax-4 spectrofluorometer (Horiba Scientific, Kyoto, Japan). Fluorescent nanoparticles and Cell Apoptosis Assay were observed with a Nikon A1R Confocal Microscope System (Tokyo, Japan). The surface tension and contact angle of the CDs, SiO_2_ and SiO_2_@CDs were determined using a high-speed optical contact angle instrument (SL200 KB, Kino Industry, Minhang, Shanghai, China). The Thermogravimetric analysis (TGA) of CDs, SiO_2_, and SiO_2_@CDs were performed using TGA 550 (Discovery, America, USA).

### Synthesis and characterization of NMs

Details of experimental methods can be found in the Supporting Information.

### Greenhouse study

Tobacco seedlings were initially grown in indoor nursery trays until they reached 6–7 true leaves. Uniform seedlings were selected and then cultured hydroponically for 10 days with a 20% Hoagland nutrient solution. The nutrient solution was refreshed every 3 days to ensure optimal conditions. After the 10-day period, the tobacco leaves were treated with a 3 mL solution of NMs using a hand-held sprayer. This experiment followed a completely randomized design, incorporating two factors: NMs treatment and aphid treatment. The sprays comprised four treatments: 100 mg/L (CDs, SiO_2_, and SiO_2_@CDs), pure water as a control (CK), aphid feeding treatment with the addition of two-day-old wingless parthenogenetic female adult aphids (+ Aphids), and aphid feeding without aphids (-Aphids). Each plant was infested with 20 aphids and covered with a 75 μm mesh bag to prevent their escape. Each treatment was replicated four times, and the plants were sprayed once daily for 7 days, with the roots covered by tinfoil during spraying. In the case of aphid infestation, the number of adult aphids present on the plants was recorded daily.

### Evaluation of the direct entomotoxic effects of NMs on aphids

Equal areas of tobacco leaf were immersed in solutions of different concentrations of NMs solution (CDs, SiO_2_ and SiO_2_@CDs), ranging from 0.1, 1, 10, 100 to 1,000 mg/L for 60 s and air-dried for 2 h. Subsequently, the treated leaves were placed on plastic bowls, and 10 two-day-old wingless parthenogenetic female adult aphids were transferred to each plastic bowl using a fine brush. The process was repeated four times for each treatment. Additionally, an equal amount of pure water was used as a control (CK). The number of adult aphids that survived was recorded at 24, 48, and 72 h, respectively.

### Effect of NMs on the activity of antioxidative enzymes in tobacco

To analyze the effects of the treatments on tobacco leaves, approximately 100 mg of the tobacco leaves was weighed, and 1mL of extraction solution was added. The mixture was then homogenized in an ice bath, followed by centrifugation at 8,500 rpm for 10 min at 4 °C. The supernatant was collected and kept on ice for subsequent measurement. The concentrations of superoxide dismutase (SOD), CAT, POD, and malondialdehyde (MDA) were determined according to a kit following the provided instructions (details in Supporting Information).

### Effect of NMs on tobacco plant’s resistance against aphids

The determination of Si was performed using the colorimetric molybdenum blue method. Initially, tobacco leaves were dried at 80 °C and then sieved. A 50 mg sample of the sieved leaves was weighed, and 1 mL of NaOH solution was added for grinding. The sample was then heated in a 95 °C water bath for 1 h, followed by cooling to room temperature and thorough shaking. After centrifugation at 25 °C for 10 min at 9,000 rpm, the supernatant was collected for analysis. Following the kit instructions, the supernatant was treated, mixed thoroughly, and allowed to stand at 25 °C for 20 min. A 200 µL aliquot of the mixture was then added to a 96-well plate, and the Si concentration was determined by recording the absorbance at 650 nm.

The levels of phytohormones (salicylic acid, SA) in tobacco leaves were assessed based on the method described in the ELISA kit instructions (refer to Supporting Information for more details).

### Metabolome and transcriptome sequencing and analysis

Leaves infested with aphids, which were either treated with 100 mg/L SiO_2_@CDs or pure water, were collected, and immediately frozen using liquid nitrogen before being stored at -80 °C. These samples were then shipped to Biomarker Technologies (Shunyi, Beijing, China) for RNA sequencing, extraction, and identification of leaf metabolites. Gene expression levels were quantified by fragments per kilobase of transcript per million fragments mapped. RNA expression levels were compared using DESeq2, DEGseq, and edgeR to identify differences. An annotation of gene function was performed by comparing Kyoto Encyclopedia of Genes and Genomes (KEGG) and Gene Ontology (GO) databases.

Methods for metabolome sequencing and analysis can be found in Supporting Information. There were 6 biological replicates in the non-target metabolome and 3 for transcriptome analysis.

### Biosafety assessment of SiO_2_@CDs in soil, water and natural enemy insects

*Nontarget biosafety in soil*. To start, 18 portions of 500 g of dried soil were prepared. Subsequently, solutions of SiO_2_@CDs NMs solutions at five concentrations (0.1, 1, 10, 100, and 1,000 mg/L) were meticulously prepared and mixed into the soil to achieve a consistent water content of approximately 25%. As a control, pure water treatment (CK) was also included in the experiment. Following the soil preparation, 20 earthworms (~ 100 mm, Jinnong No. 6) were introduced in each treatment group. The containers housing the treatments were then sealed with gauze and covered with a perforated lid before being placed in an incubator. Throughout the experiment, the temperature was maintained at 20 ± 2 °C, relative humidity of 80 ± 10%, and complete darkness was ensured within the incubator. On both the 7th and 14th days of the experiment, the earthworms were carefully removed from each treatment group, and any deaths were promptly recorded. This precise methodology was employed to assess the impact of SiO_2_@CDs NMs on the earthworm populations under controlled environmental conditions.

*Nontarget biosafety in water*. Acute toxicity testing was conducted on blue zebrafish (*D. rerio*) in accordance with OECD 203 guidelines [43]. Fifty blue zebrafish measuring 2.5 ± 0.5 cm (in length) were acclimated in chlorine-free water under a 12 h light exposure cycle. Ten blue zebrafish were then exposed to different concentrations (0.1, 1, 10, 100, and 1,000 mg/L) of SiO_2_@CDs NMs, while a control group (CK) was treated with pure water. The mortality of the zebrafish in each treatment group was recorded at 24, 48, 72, and 96 h.

*Biosafety to natural enemy insects.* The filter paper contact method was used for the determination [44]. Different concentrations (0.1, 1, 10, 100, and 1,000 mg/L) of SiO_2_@CDs NMs were prepared in pure water and carefully dropped on a filter paper-lined Petri dish (15 cm in diameter). Ten adult ladybirds were placed into each Petri dish to freely crawl and come into contact with the NMs, with pure water used as a control. After 2 h of exposure, *H. variegate* adults were transferred individually to Petri dishes containing aphids for feeding at 25 °C. The number of dead ladybirds was recorded at 24, 48, and 72 h. *H. variegate* that remained still when touched with a brush were classified as dead.

### Biosafety to human cells

*Cell viability assay.* During the logarithmic growth phase, L02 cells were trypsin-digested and seeded into 96-well plates at 6 × 10^3^ cells/well. In the control group, 100 µL of complete medium was added, while in the sample group, 100 µL of the SiO_2_@CDs working solution (at a concentration of 0, 0.5, 1, 5, 10, 50, 100, 500, and 1,000 mg/L) was added. The plates were then placed in an incubator at 37 °C with 5% CO_2_ for 24 h. Following incubation, the medium was discarded, and the wells were washed three times with PBS. Subsequently, 120 µL of fresh medium containing 10% CCK-8 was added to each well, and the plates were returned to the incubator at 37 °C for an additional 2 h. After incubation, 100 µL of the supernatant from each well was transferred to a new plate, and the absorbance was measured at 450 nm using an enzyme reader to determine cell viability based on the following formula:

$$\text{cell viability}= \frac{\text{OD}_\text{treatment group}-\text{OD}_\text{blank group}}{\text{OD}_\text{control group}-\text{OD}_\text{blank group}} \times 100\% $$  

*Cell apoptosis assay.* L02 cells, in their logarithmic growth phase, were seeded in 6-well plates at a density of 4 × 10^5^ cells per well and incubated at 37 °C with 5% CO_2_ for 24 h to allow cell attachment. SiO_2_@CDs were then added and cultured for an additional 24 h. Subsequently, cells were digested, centrifuged, and resuspended in cold buffer, followed by staining with PI/FITC Apoptosis Kit for 15 min in the dark. Apoptosis was then evaluated using flow cytometry.

For laser confocal imaging, L02 cells were seeded at 8 × 10^4^ cells/well in confocal dishes and allowed to adhere overnight. Following 24 h of culturing with control and sample groups, the cells were washed with PBS and stained with PI/Calcein-AM for 15 min in the dark. Images were then captured using laser confocal microscopy.

### Statistical analysis

All statistical analyses were conducted using IBM SPSS Statistics 27 (Chicago, IL, USA). After assessing the normality and variance homogeneity of data through Shapiro-Wilk’s test. As the results indicated that certain datasets deviated from a normal distribution, appropriate transformations (natural logarithm, ln) were applied prior to ANOVA to satisfy its assumptions. Two-way ANOVAs were used to evaluate the effects of NMs and aphid treatments on tobacco growth characteristics, antioxidant enzyme activities, Si, and phytohormone levels. Following this, one-way ANOVAs were used to examine the impact of NMs on aphid populations, both in the presence and absence of aphids. Tukey’s HSD post hoc test was used to determine treatment differences at *P* < 0.05. All results are presented as means ± standard errors (SEs), with a sample size of *n* = 4 for each group.

## Result and discussion

### Characterization of NMs

**Fig. 1:**
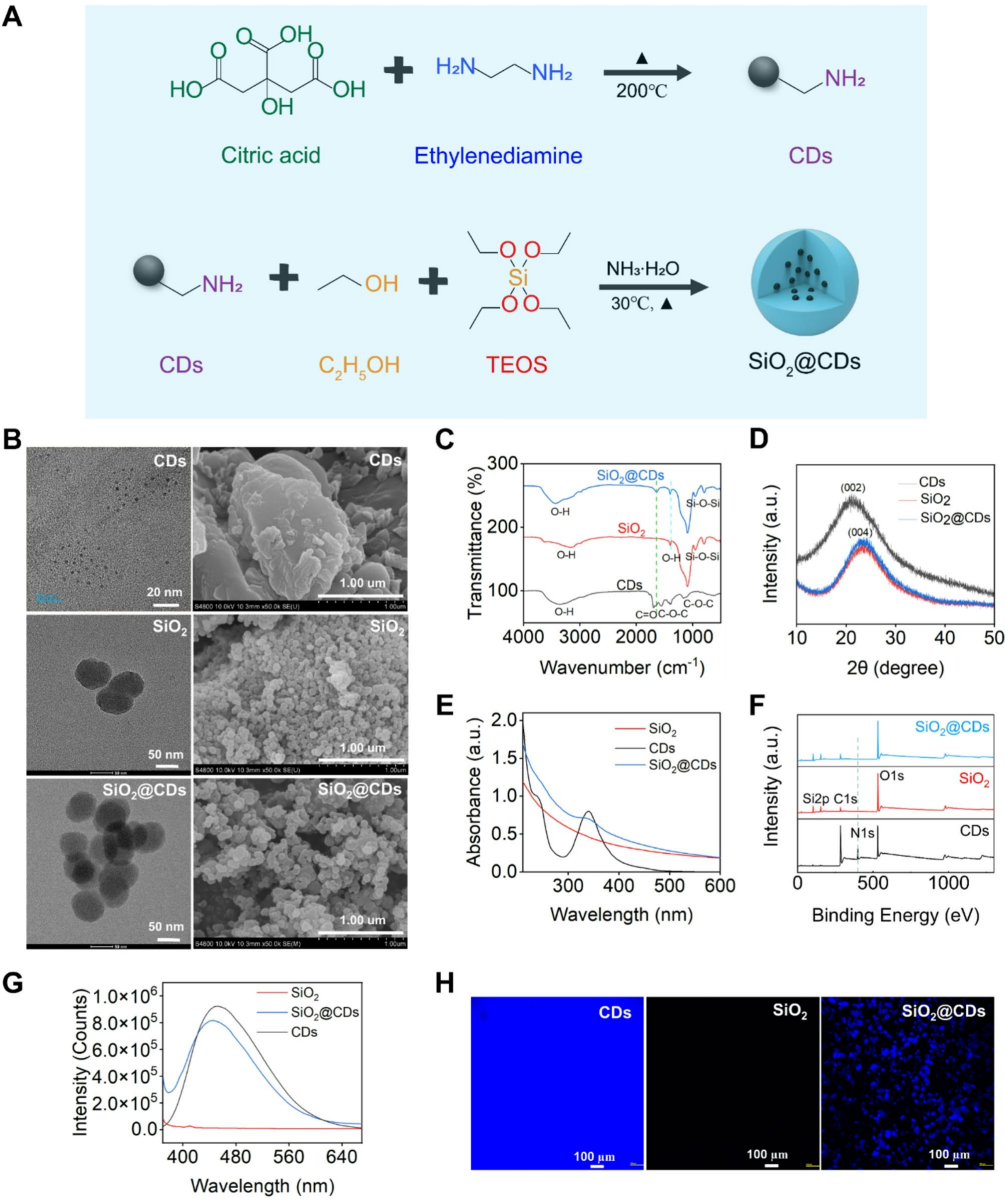
The correlative characterization of CDs, SiO_2,_ and SiO_2_@CDs. (**A**) Schematic illustration of the preparation process for SiO_2_@CDs; (**B**) TEM and SEM images; (**C**) FT-IR spectra; (**D**) XRD patterns; (**E**) UV-vis absorption spectra; (**F**) XPS spectrum; (**G**) Fluorescence curves; (**H**) Confocal laser scanning microscope of CDs, SiO_2_, and SiO_2_@CDs

Initially, CDs were synthesized through a high-temperature reaction with citric acid, and then SiO_2_@CDs composites were prepared through a sol-gel method (Fig. 1A). Subsequent characterization tests were conducted to verify the successful formation of SiO_2_@CDs. Both SEM and TEM were utilized to analyze the CDs, SiO_2_, and SiO_2_@CDs NMs (Fig. 1B) revealing uniform spherical particles for both CDs and SiO_2_. Statistical analysis performed using Nano Measurer software determined the average particle sizes of CDs and SiO_2_ to be 1.71 nm (Figure S1) and 65 nm, respectively. While SiO_2_@CDs also appeared a spherical morphology, the HRTEM image clearly reveals the embedding or encapsulation of CDs within the SiO_2_ structure, providing further evidence of the successful loading of CDs onto SiO_2_ (Figure S2A). The successful synthesis of SiO_2_@CDs was further verified through FT-IR spectrum (Fig. 1C). The peaks observed at 3465 cm^-1^ and 1644 cm^-1^ in the FT-IR spectrum of CDs are attributed to the stretching vibrations of O-H and C = O bonds, respectively. These findings suggest that the surface of CDs is rich in hydroxyl and carboxyl groups. Additionally, the bands at 1413 cm^-1^ and 1160 cm^-1^ are attributed to the asymmetric and symmetric stretching vibrations of the C-O-C linkage, respectively [45]. In contrast, the FT-IR spectrum of SiO_2_ shows typical absorption bands, including the asymmetric stretching vibration of Si-O-Si observed at 1100 cm^-1^, the Si-O stretching vibration appears at 966 cm^-1^, and the Si-O-Si symmetric stretching vibration at 810 cm^-1^. In the FT-IR spectrum of SiO_2_@CDs, in addition to the characteristic peaks of SiO_2_, the absorption peak at 1644 cm^-1^ corresponding to the C = O stretching vibration further confirms the presence of CDs, indicating the successful encapsulation of CDs by SiO_2_. This was further confirmed by verification of the crystalline structure of the composite material using the XRD (Fig. 1D). The CDs exhibited a prominent broad peak at around 2θ = 20°, indicative of the presence of the (002) crystallographic plane of graphitized carbon, suggesting some degree of graphitization. However, the peak’s broadness suggests a lack of highly ordered structure, potentially containing amorphous carbon. On the other hand, both SiO_2_ and SiO_2_@CDs displayed a broad peak at around 2θ = 22°, indicating that the retention of the SiO_2_’s amorphous nature post-loading of CDs, albeit with some influence from CDs without significant alternation of the overall peak shape [46, 47]. Subsequently, the UV-vis absorption properties of CDs, SiO_2_, and SiO_2_@CDs were analyzed (Fig. 1E). CDs showed UV absorption peaks at 230 and 340 nm, corresponding to π → π* transitions (C = C) and n → π* transitions (C = N, C = O), respectively. While SiO_2_ did not have a characteristic peak, the combination of CDs and SiO_2_ in SiO_2_@CDs still retains the characteristic 340 nm peak seen in CDs.

Figure 1F illustrates the analysis of the chemical composition of the sample surface using three full XPS spectra. The SiO_2_ and SiO_2_@CDs samples exhibit Si2p peaks at 103 eV, confirming the presence of Si on their surface. In contrast, the CDs sample displays a distinct N1s peak at 399 eV, attributed to the amino groups from ethylenediamine retained on the surface during synthesis. Significantly, the Si2p and N1s peaks are both evident in the SiO_2_@CDs sample, indicating the successful formation of the composite material (Figure S2B). Furthermore, the fluorescence properties of the materials were utilized for verification. The SiO_2_@CDs NMs exhibited a strong fluorescence characteristic peak at 440 nm when excited at 360 nm, consistent with the typical fluorescence of CDs (Fig. 1G). Additionally, fluorescence confocal microscopy was used for visualization. CDs emit a distinct blue fluorescence, while SiO_2_ does not show a fluorescence signal. However, upon loading the CDs onto the SiO_2_, a noticeable distribution of blue fluorescence is observed in various regions (Fig. 1H). Overall, these characterization results clearly and conclusively confirm the successful preparation of SiO_2_@CDs composite.

### Superior wettability, dispersion and photodegradation resistance of NMs

**Fig. 2:**
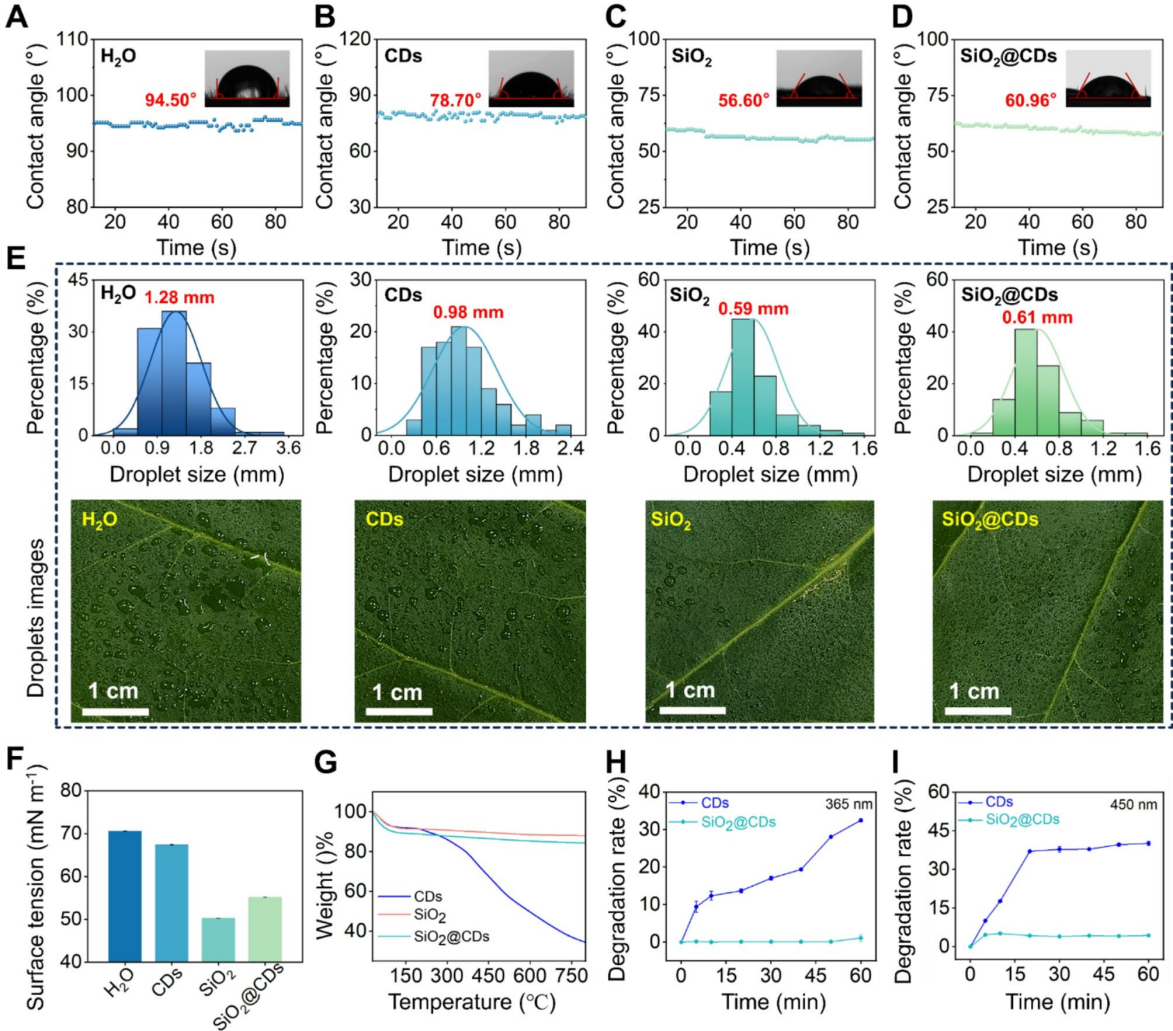
Wettability, dispersion and photodegradation resistance of NMs. (**A**-**D**) Contact angles of water, CDs, SiO_2_ and SiO_2_@CDs on tobacco at 100 mg/L; (**E**) Spraying simulation of water, CDs, SiO_2_, and SiO_2_@CDs on tobacco at 100 mg/L; (**F**) The surface tension measurements of water, CDs, SiO_2_, and SiO_2_@CDs; (**G**) The TGA curves of CDs, SiO_2_, and SiO_2_@CDs; The photostability of CDs and SiO_2_@CDs under 365 nm (**H**) and 450 nm (**I**) excitation

In practical applications, it is essential for SiO_2_@CDs to achieve effective wetting and dispersion on leaf surfaces when applied through foliar spraying. This is crucial for promoting material utilization efficiency. To assess the performance of NMs on tobacco leaves, various tests such as contact angle measurement, surface tension analysis, and droplet spray testing were conducted. The results revealed that the average contact angle of water droplets on tobacco leaves was approximately 94.50°, indicating the hydrophobic nature of the leaf surface (Fig. 2A**)**. The contact angles of CDs and SiO_2_ on the foliar surface were 78.70° and 56.60°, respectively (Figs. 2B-C). Interestingly, the contact angle of SiO_2_@CDs on tobacco leaves was found to be 18° lower than that of CDs alone (Fig. 2D). The low contact angle indicates that the SiO_2_ has good affinity with the leaf surface. As a result, the SiO_2_@CDs composite materials exhibit improved contact with water on the leaf surface compared to pure CDs alone, leading to a reduced contact angle [48]. This finding highlights the enhanced performance and potential of SiO_2_@CDs for practical applications.

To visually investigate the dispersion performance of the materials, the NMs solutions were sprayed onto tobacco leaves under controlled conditions (Fig. 2E). On the leaf surface, water droplets appeared in large, unevenly spaced clusters, with an average droplet size of 1.28 mm. In contrast, the droplets in the CDs and SiO_2_-treated groups were significantly smaller, with average sizes of 0.98 mm and 0.59 mm, respectively, and showed a more uniform distribution. Remarkably, the average droplet size on leaves treated with SiO_2_@CDs was 0.61 mm, similar to the droplet size seen on leaves treated with SiO_2_ alone. This observation can be attributed to the negative charge carried by SiO_2_, which aids in reducing particle aggregation and promotes better dispersibility by mimicking inter-particle attraction (the SiO_2_ potential diagram is shown in Figure S3). These findings were supported by the surface tension test results presented in Fig. 2F. Compared to the surface tension of CDs solution (67.45 mN/m), the surface tension of the SiO_2_@CDs significantly decreased to 55.18 mN/m. This indicates that its droplets of SiO_2_@CDs exhibit superior wetting and dispersion properties on leaf surfaces. The thermal stability and decomposition behavior of SiO_2_@CDs were investigated using TGA (Fig. 2G). In the temperature range of 100 °C to 800 °C, the weight of CDs decreased significantly, with a sharp decline beginning at around 400 °C, indicating decomposition or oxidation at high temperatures. In contrast, SiO_2_ exhibited high thermal stability throughout the entire temperature range, with minimal weight change, demonstrating excellent thermal stability. For the SiO_2_@CDs composite, the weight loss fell between that of pure SiO_2_ and pure CDs, indicating enhanced stability compared to pure CDs. The presence of SiO_2_ provided a protective effect, enhancing the thermal stability of the CDs. The main weight losses of SiO_2_@CDs and SiO_2_ were 11.8% and 15.35%, respectively, allowing for the determination of the CDs content in the SiO_2_@CDs composite to be 3.55%.

In order to further assess the photostability of CDs and address concerns about their tendency to generate excessive ROS under light exposure, potentially causing damage to cells and organisms, we conducted a study on SiO_2_@CDs. Our results, as shown in Fig. 2H and I, demonstrate that after 1 h of irradiation under UV light (365 nm) and visible light (450 nm), the degradation rates of CDs were 32.51% and 40.10%, respectively. Contrastingly, the degradation rates of SiO_2_@CDs under the same conditions were significantly lower, at only 1.11% and 4.39% for UV and visible light, respectively. This significant difference highlights the protective effect of the SiO_2_ coating, which substantially improves the photostability of CDs and enhances their biocompatibility. Furthermore, the blue fluorescence of CDs allowed us to visually observe the uptake and transportation of SiO_2_@CD in tobacco leaves. Confocal imaging revealed a distinct blue color in the tobacco leaves treated with SiO_2_@CD, as compared to the control group (CK) treated with pure water (Figure S4). This observation indicates that successful absorption and transportation of SiO_2_@CD by the tobacco plant, showcasing its potential as an efficient delivery system.

### Effect of supplemental spraying treatment on tobacco seedlings

**Fig. 3:**
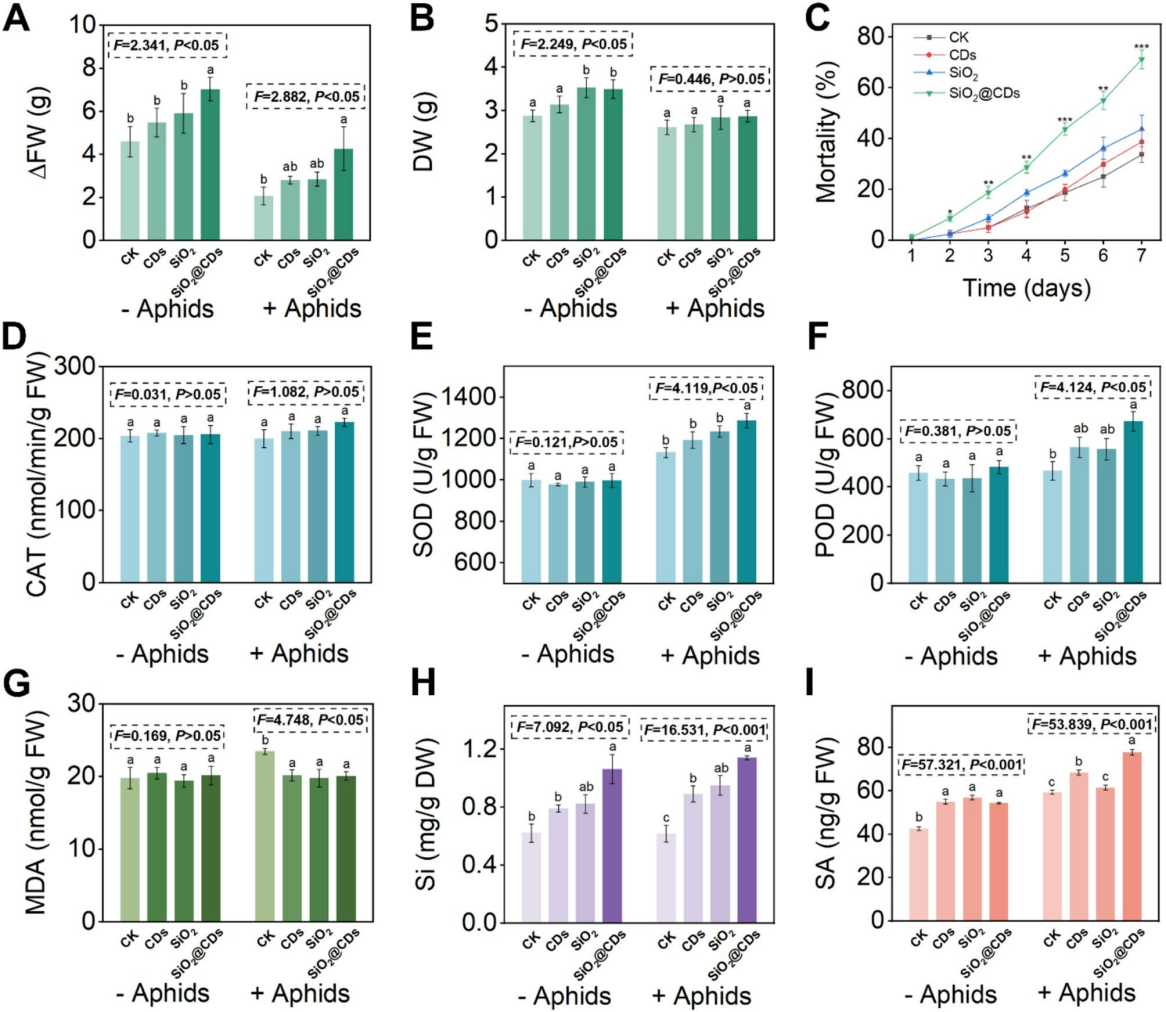
Effects of supplemental spraying treatments of NMs on tobacco growth, aphid mortality, and plant resistance responses. (**A**) ΔFW and (**B**) DW of tobacco after CK, CDs, SiO_2_, and SiO_2_@CDs exposure (ΔFW= FW_(7 days after feeding treatment)_ - FW _(0 days after feeding treatment)_); (**C**) The effect of CK and NMs (CDs, SiO_2_, SiO_2_@CDs) on the number of adult aphids 1–7 days after aphid infestation; (**D**–**I**) Leaf (**D**) CAT, (**E**) SOD, (**F**) POD, (**G**) MDA, (**H**) Si and (**I**) SA of tobacco without (− Aphids) and with (+ Aphids) the presence of aphids exposed to CK, CDs, SiO_2_ and SiO_2_@CDs. Data are presented as means ± SD (*n* = 4). Statistical analysis was performed using one-way ANOVA followed by Tukey’s multiple comparison test. Significant differences were observed among treatments, and *F*- and *P*-values are indicated in each panel

Research has shown that SiO_2_ NMs or CDs have the potential to increase the biomass of *Glycine max* or mung bean. In this particular study, a significant impact of SiO_2_@CDs on the growth of tobacco plants was identified, both in the presence or absence of aphids (Figs. 3A-B). The combined impact of NMs and herbivore treatments did not significantly affect the change in fresh weight (ΔFW) and dry weight (DW) (Table S1). The one-way ANOVA showed that the different treatments had a significant effect on the ΔFW of tobacco under both aphid-free and aphid-infested conditions (− Aphids: *F* = 2.341, *P* < 0.05; +Aphids: *F* = 2.882, *P* < 0.05). Under aphid-free conditions, SiO_2_@CDs significantly increased the DW of tobacco plants compared to CK and CDs treatments (*F* = 2.249, *P* < 0.05). However, no significant difference was observed among treatments under aphid infestation (*F* = 0.446, *P* > 0.05). Specifically, under aphid-free conditions (− Aphid), the application of SiO_2_@CDs resulted in a substantial increase of 53% in tobacco ΔFW and a 21% increase in DW compared to the control. Furthermore, under aphid infestation, SiO_2_@CDs still promoted plant growth compared to the CK group, indicating that their beneficial effects were maintained under biotic stress conditions. Altogether, these results confirm that the photosynthetic advantages associated with CDs were preserved upon incorporation into SiO_2_, contributing to the observed positive effects on plant growth, even under biotic stress conditions. As shown in Fig. 3A and B, SiO_2_@CDs exhibited comparable or even superior growth-promoting effects to CDs under both aphid-free and aphid-infested conditions. Additionally, we conducted measurements of net photosynthetic rate (Pn) and chlorophyll content (SPAD) (Figure S5). The results revealed no significant differences between the SiO_2_@CDs and CDs treatments for both parameters, indicating that the photosynthesis-enhancing ability of CDs was preserved after loading.

### Enhanced resistance of tobacco to aphid infestation by SiO_2_@CDs

After recognizing the excellent physicochemical properties and wetting-dispersing capabilities of SiO_2_@CDs, we proceeded with a study to evaluate their effectiveness in enhancing tobacco resistance to aphids. To ensure that aphids were not directly harmed by the NMs, we initially conducted an in vitro insecticidal activity test using a leaf-dipping method. The results showed that, across all treatment groups (including three materials: CDs, SiO_2_ and SiO_2_@CDs; five concentrations: 0.1, 1, 10, 100, and 1,000 mg/L, and a control (CK)), there were no significant effects on the number of adult aphids 1–3 days after infestation, with the exception of the high concentration treatment (1,000 mg/L) (Figure S6). Moreover, at concentrations of 100 mg/L or lower, none of the NMs treatments (CDs, SiO_2_ and SiO_2_@CDs) showed a significant decrease in the number of adult aphids at 24, 48, and 72 h post-treatment when compared with the CK, indicating that low-doses of NMs did not cause direct toxicity to the aphids. Based on these findings, we selected a dose of 100 mg/L of NMs for foliar spray application on tobacco plants to assess their potential in enhancing aphid resistance. Remarkably, when tobacco plants were treated with NMs, the SiO_2_@CDs treatment group demonstrated a significant reduction in the number of adult aphids 2 days after infestation (Fig. 3C). After 7 days of infestation, the number of adult aphids in the SiO_2_@CDs treatment group was reduced by 71%, which was significantly higher than the reductions observed in the with CDs (39%) and SiO_2_ (44%) treatments. These results indicate that SiO_2_@CDs are the most effective in controlling aphid reproduction, potentially due to the synergistic effects of CDs and SiO_2_ in activating plant resistance (the images of aphid-infested tobacco plants are shown in Figure S7). Specifically, SiO_2_ contributes to strengthening the plant’s physical barriers (e.g., thickened cell walls and enhanced cuticle), whereas CDs trigger endogenous biochemical defense responses. This interplay between physical and biochemical defenses facilitates a faster and more robust resistance against aphid attacks, thereby providing both immediate and long-lasting protection [29, 49].

### SiO_2_@CDs enhanced the resistance mechanism of tobacco to aphid infection

To further investigate how SiO_2_@CDs enhance tobacco resistance to aphids, we systematically evaluated the antioxidant defense mechanisms in aphid-stressed tobacco. We assessed the activity levels of three essential antioxidant enzymes—CAT, SOD, and POD—and determined the concentration of MDA, a key indicator of lipid peroxidation (Fig. 3D and G). The results showed that CAT activity (Fig. 3D) remained consistent across all treatment groups (–Aphids: *F* = 0.031, *P* > 0.05; +Aphids: *F* = 1.082, *P* > 0.05), regardless of aphid infestation, indicating that CAT may not be the primary scavenging enzyme in tobacco for active ROS under our experimental conditions. Under aphid-free conditions, no significant differences were observed in SOD and POD activities among treatments (*P* > 0.05). In contrast, aphid infestation significantly increased SOD activity in the SiO_2_@CDs treatment group (*F* = 4.119, *P* < 0.05), while POD activity exhibited an increasing trend (*F* = 4.124, *P* < 0.05), though not statistically distinct from other treatments. However, SOD (Fig. 3E) and POD (Fig. 3F) activities rose by 14% and 44%, respectively, following SiO_2_@CDs treatment. This suggests that in the SiO_2_@CDs treatment group, SOD and POD activities were observed. This indicates that SiO_2_@CDs effectively enhance the antioxidant capacity of tobacco by reducing oxidative damage through ROS scavenging. MDA is a terminal product of membrane lipid peroxidation, and its excessive accumulation can lead to the cross-linking and polymerization of proteins, lipids, and nucleic acids, negatively impacting plant growth and development. Our study found that under aphid stress, compared to the CK group, the CDs, SiO_2_ and SiO_2_@CDs treatments significantly reduced MDA content in tobacco leaves by 14%, 16%, and 15%, respectively (Fig. 3G) (*F* = 4.748, *P* < 0.05). These results demonstrate that SiO_2_@CDs significantly activate SOD and POD, enhancing the antioxidant defense in tobacco plants. By reducing MDA accumulation, they alleviate lipid peroxidation induced by aphid-induced stress, thereby improving tobacco resistance. SiO_2_@CDs-induced enhancement may be attributed to their improved wettability and foliar adhesion, as well as the combined effect of CDs and SiO_2_ in simultaneously activating both physical and biochemical defense mechanisms in plants [50–53].

Further analysis revealed that the application of CDs, SiO_2_ and SiO_2_@CDs resulted in a substantial increase in Si content in aphid-stressed tobacco leaves, with percentages of 44%, 54%, and 85%, respectively (Fig. 3H) (*F* = 16.531, *P* < 0.001). Notably, SiO_2_@CDs proved to be the most effective in promoting Si accumulation in tobacco compared to the other components. The enhanced Si content may be contributed to the promotion of silicic acid (H_4_SiO_4_) production by their application of SiO_2_@CDs [54]. Moreover, it has been well-documented that phytohormones, such as SA, play a dominant role in plant defense mechanisms against herbivores. Our study found that both NM treatments and aphid infestation had a significant impact on SA level in tobacco leaves, and their interactions led to a synergistic effect (Table S1). SA levels differed significantly among treatments under both aphid-free and aphid-infested conditions. Following a 7-day period of aphid stress, it was observed that CDs, SiO_2_, and SiO_2_@CDs treatments resulted in a significantly increased SA content in tobacco leaves by 15%, 4%, and 31%, respectively (Fig. 3I). This is consistent with previous findings that Si enhances SA accumulation in plants under biotic stress [55]. When compared to CDs and SiO_2_ treatments SiO_2_@CDs exhibited better protection in both physical and chemical defense mechanisms in plants, likely due to the combined effect of CDs and nano-SiO_2_. This composite integrates the structural stability of SiO_2_ with the biological activity of CDs, significantly enhancing plant resistance to aphid infestation. SiO_2_ promotes cell wall thickening and silica deposition, thereby strengthening physical barriers, while CDs activate endogenous immune responses and induce biochemical defenses. In addition, SiO_2_@CDs markedly increased the activities of antioxidant enzymes such as SOD and POD, which contribute to the scavenging of ROS induced by aphid feeding, alleviating oxidative stress and indirectly suppressing aphid survival and reproduction [56–58]. In conclusion, the enhanced entomotoxicity of SiO_2_@CDs does not result from a single material property but arises from the coordinated activation of multiple plant defense pathways—including redox regulation, phytohormone signaling, and physical barrier enhancement.

### Leaf metabolic and molecular responses to SiO_2_@CDs application

**Fig. 4:**
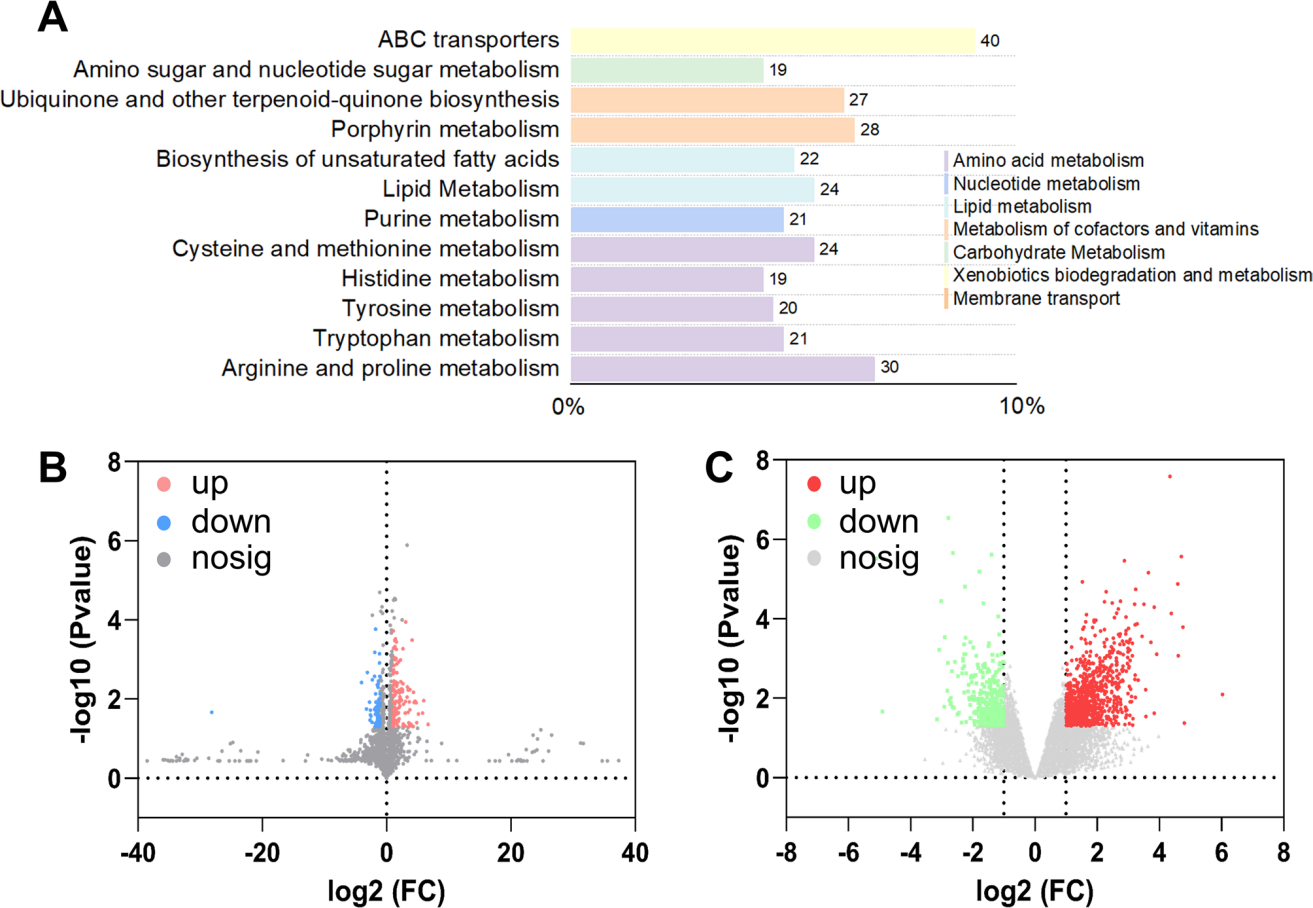
Metabolomic and transcriptomic responses of tobacco leaves to SiO_2_@CDs treatment under aphid stress. (**A**) Summary of metabolite KEGG database annotation categories; (**B**) Volcano plot of differential metabolites; (**C**) Volcano plot of differential gene expression

To further investigate the potential of SiO_2_@CD to induce defense responses beyond the physical and chemical defenses mentioned above and to enhance tobacco leaf resistance to aphids, we conducted transcriptomics and metabolomics analyses. In this study, we further analyzed the non-targeted metabolomics of tobacco leaves under aphid stress, finally identified 3,856 metabolites. All identified metabolites were annotated using the KEGG database, and the top 20 KO pathway level 3 entries with the most annotations were selected. A summary bar chart was generated to illustrate the metabolic pathways related to tobacco (Fig. 4A). A total of 430 differentially abundant metabolites were detected, among which the levels of 159 were reduced and those of 271 were increased (Fig. 4B; Table 1). These differentially abundant metabolites were primarily categorized into 9 classes, which include secondary metabolism, stress adaptation, and energy metabolism (Figure S8). Furthermore, a total of 571 DEGs were found from this transcriptome, accounting for 2.38% of the total annotated genes (23,946) (Fig. 4C; Table 1), of which 165 genes were downregulated and 406 were upregulated. Based on sequence homology, 571 DEGs were annotated to 25 functional categories, including 14 biological processes, 3 cellular components, and 8 molecular functions (Figure S9). KEGG pathway enrichment analysis shown in Figure S10 indicates that SiO_2_@CDs treatment significantly influenced plant hormone signal transduction, photosynthesis, sugar metabolism, energy metabolism, and stress response, all of which are crucial for exploring plant metabolic regulation and environmental adaptability.

**Table 1:** Statistical table of differential metabolites and differentially expressed genes

Group	Category	Total-num	Diff-num	Up-num	Down-num
CK vs SiO_2_@CDs	Metabolites	3,856	430	271	159
	Expressed genes	23,946	571	406	105

(Group: Information on differential metabolite/gene grouping. Total-num: Total number of identified substances. Diff-num: Number of significantly different metabolites/genes. Up-num: Number of upregulated metabolites/genes. Down-num: Number of downregulated metabolites/genes.)

**Fig. 5:**
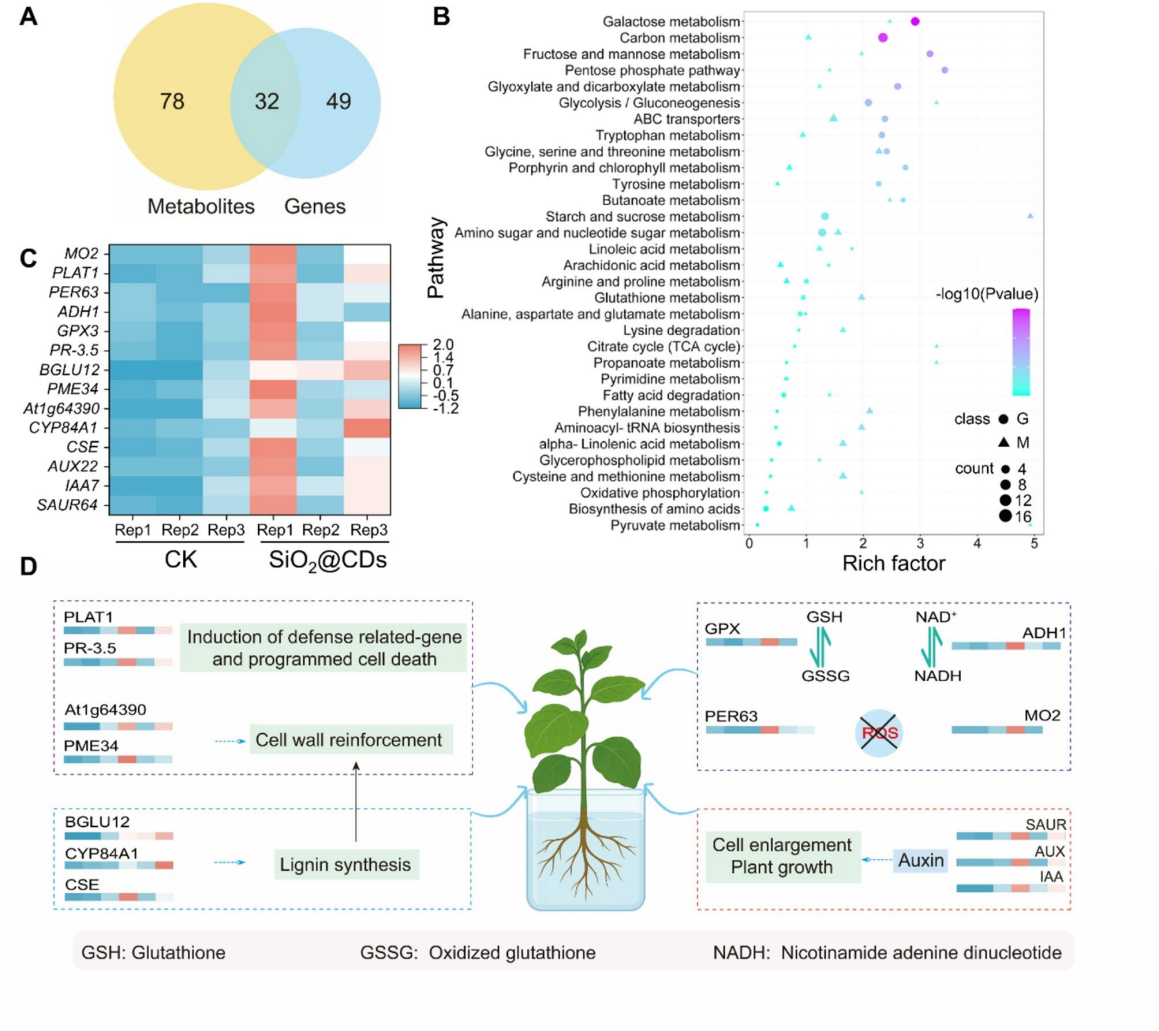
Leaf metabolic responses to SiO_2_@CDs application. (**A**) Venn diagram showing the number of transcriptomes and metabolomes gaining co-participation in pathways; (**B**) Bubble mapping of KEGG pathways co-enriched by transcriptomics and metabolomics; (**C**) The expression profile of genes in tobacco leaves treated with or without SiO_2_@CDs; (**D**) A diagram illustrating the molecular mechanisms underlying the enhancement of tobacco resistance to aphids by SiO_2_@CDs (*P* < 0.05, *n* = 3)

A Venn diagram was generated to compare the pathways involved in genes from the transcriptome and metabolites from the metabolome, highlighting the number of pathways that co-participate (Fig. 5A). We identified 78 pathways unique to metabolites, 49 pathways unique to genes, and 32 pathways shared between both. This suggests potential interrelationships or influences between metabolite and gene expression. Bubble plots of KEGG (Fig. 5B) pathways co-enriched by transcriptomics and metabolomics revealed significant gene enrichment in Galactose metabolism (ko00052), Pentose phosphate pathway (ko0003), Carbon metabolism (ko01200), Fructose and mannose metabolism (ko00051), and Glyoxylate and dicarboxylate metabolism (ko00630). On the other hand, metabolites were primarily enriched in Starch and sucrose metabolism (ko00500), Glycine (ko00360), Serine and threonine metabolism (ko00260), Phenylalanine metabolism (ko00360), Glutathione metabolism (ko00480), and Aminoacyl-tRNA biosynthesis (ko00970). These results suggest that treatment with SiO_2_@CDs enhances tobacco leaf development through energy metabolism and amino acid biosynthesis while also helping the plant respond to aphid challenges through antioxidant responses and regulation of protein synthesis (the enrichment analysis of genes and metabolites in KEGG pathways are shown in Figure S11).

After exposure to SiO_2_@CDs, there was a significant upregulation of genes related to auxin signal transduction in tobacco, including *SAUR64*, *IAA7*, and *AUX22* (*P* < 0.05). This upregulation is consistent with the well-known function of auxin in promoting plant cell expansion and growth. Furthermore, the increased biomass observed after aphid infestation following SiO_2_@CDs pre-treatment is supported by the upregulation of indole-3-acetic acid (IAA)-related genes. Moreover, exposure to SiO_2_@CDs at a concentration of 100 mg/L led to the upregulation of genes associated with cell wall reinforcement (*Atg64390*, *PME34*) and the induction of programmed cell death (*PLAT1*, *PR-3.5*) (Fig. 5C and **Table S2-3**). The enhancement in lignin synthesis suggests that SiO_2_@CDs (100 mg/L) can not only boost the physical defense mechanisms of tobacco leaves but also enhance the plant’s capacity to mitigate ROS generated by aphid infestation. In the glucose metabolism pathway, genes related to polygalacturonase, sucrose, and glucose synthesis (*CSE*, *CYP84A*, and *BGLU12*) were also found to be upregulated (*P* < 0.05). These substances contribute to fructose synthesis, which indirectly promotes lignin formation and directly supports tobacco growth. Furthermore, analysis of antioxidant-related genes showed that the peroxidase-related genes *PER63*, *MO2*, *ADH1*, and *GPX3* were upregulated. Si plays a critical role in regulating ROS levels, strengthening cell wall structures, generating anti-insect secondary metabolites, and transmitting defense signals in plant defense mechanisms, with peroxidase being a key player in these processes (Fig. 5D). The upregulation of specific genes not only helps plants combat aphid damage but also strengthens their overall resistance against various biotic stresses. Following treatment with SiO_2_@CDs, the upregulation of these genes showcases the intricate nature of plant defense mechanisms in response to aphid attacks. These genes play a role in fortifying plants against aphid invasion by enhancing the stability of cell membranes and cell walls, activating defense signaling pathways, promoting energy metabolism, and accelerating damage repair. Essentially, the findings from transcriptional and metabolomics analyses indicate that SiO_2_@CDs enhance tobacco growth and resistance through the following mechanisms: (1) improving carbon metabolism and auxin signal transduction to promote plant growth; (2) enhancing lignin synthesis and reinforcement of the cell wall; (3) stimulating programmed cell death for activating self-defense mechanisms in tobacco; and (4) boosting the capacity to eliminate ROS.

### Biosafety assessment of SiO_2_@CDs

**Fig. 6:**
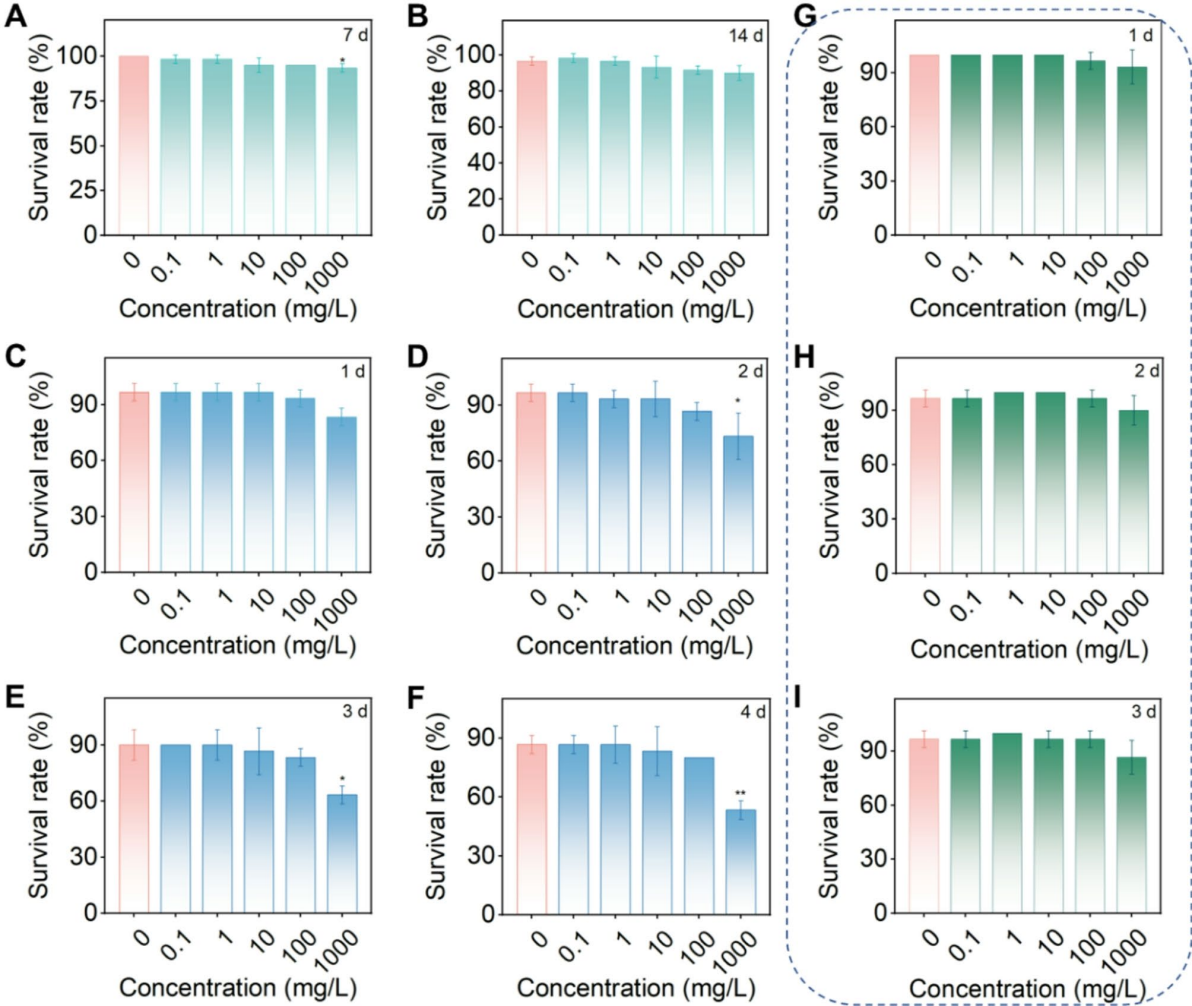
Biosafety assessment of SiO_2_@CDs. Acute toxicity test of SiO_2_@CDs toward (**A**-**B**) earthworms, (**C**-**F**) zebrafish and (**G**-**I**) ladybirds (*P < 0.05, *n* = 3)

In this section, we conducted a comprehensive evaluation of the environmental safety of SiO_2_@CDs. Earthworms (*E. fetida*) and blue zebrafish (*D. rerio*) were chosen as representative bioindicator species for soil ecosystems and aquatic environments, respectively, to conduct acute toxicity assessments and determine whether SiO_2_@CDs pose potential ecological risks. The results shown in Fig. 6A and B indicated that earthworms treated with SiO_2_@CDs at concentrations ranging from 0.1 to 1,000 mg/L exhibited survival rates above 90% after 7 and 14 days of observation. According to toxicity classification criteria, SiO_2_@CDs were classified as low toxicity to earthworms. Figure 6C and F display the survival rates of zebrafish treated with various concentrations of SiO_2_@CDs over 1 to 4 days. Zebrafish in all treatment groups showed a survival rate above 80% at concentrations between 0.1 and 100 mg/L, indicating low toxicity. We also evaluated the toxicity of SiO_2_@CDs to ladybirds (*H. variegate*), which are natural predators of aphids in agricultural ecosystems. The results in Fig. 6G and I show that survival rates of adult ladybirds treated with various concentrations of SiO_2_@CDs ranged from 97 to 100% at the lowest concentration (0.1 mg/L) and remained at 87% even at the highest concentration (1,000 mg/L). There was no significant difference (*P* > 0.05) compared to the control group (the treatment with pure water, at a concentration of 0), suggesting that SiO_2_@CDs are safe for beneficial organisms. In conclusion, SiO_2_@CDs exhibit strong environmental friendliness, showing high safety for aquatic and soil organisms as well as beneficial predators.

### Cytotoxicity assessment of SiO_2_@CDs

**Fig. 7:**
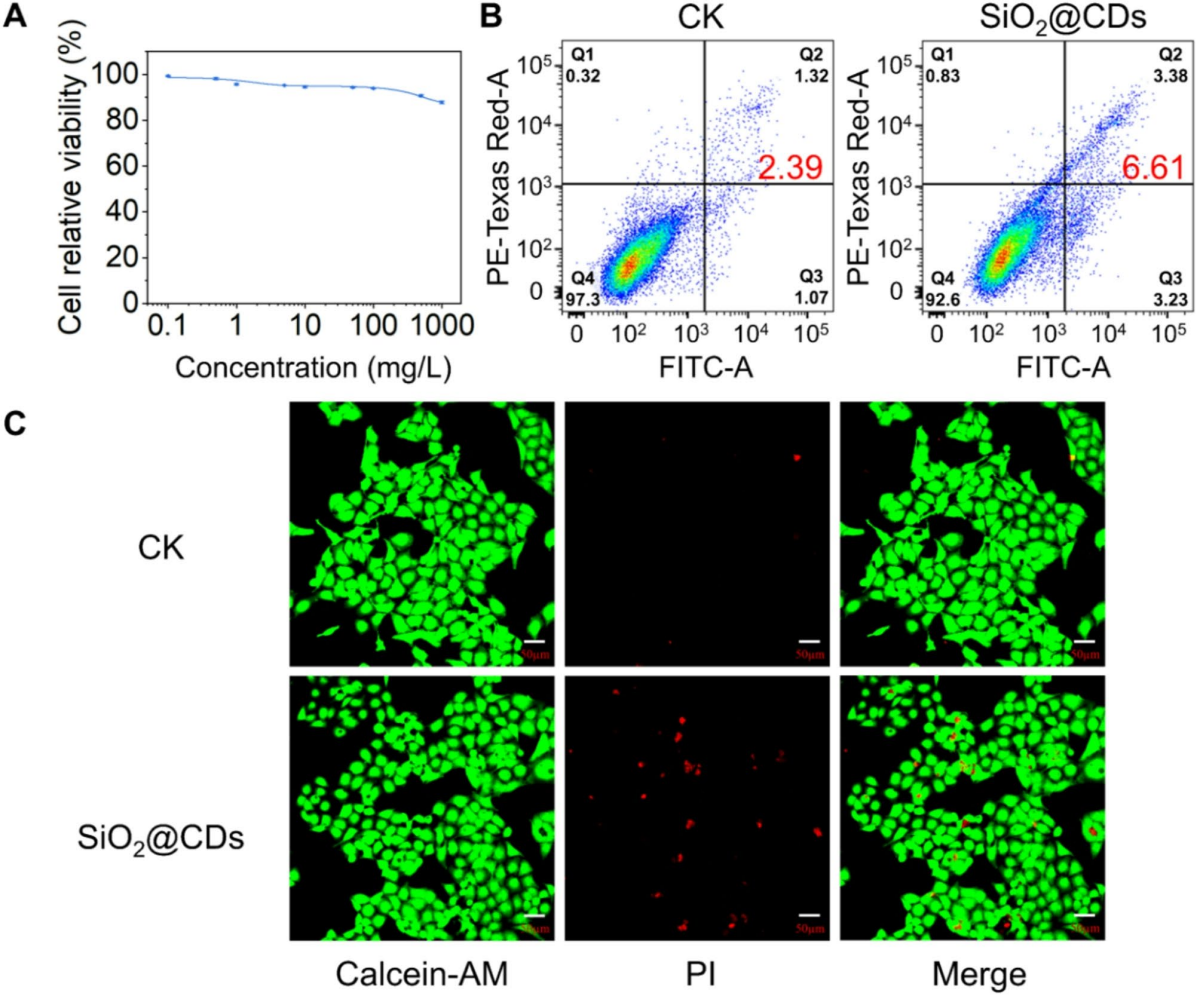
Cytotoxicity of SiO_2_@CDs. Results of the (**A**) CCK-8 assay and (**B**) flow cytometry analysis; (**C**) Images of living/dead cell staining (*n* = 3)

In practical applications, pesticides are primarily applied through spraying, which can increase the risk of them entering the human body. Once pesticides enter the body, they are quickly metabolized and can accumulate, particularly in the liver, potentially posing health risks [59]. This study focused on evaluating the cytotoxicity of SiO_2_@CDs using human L02 liver cells. The CCK-8 assay was employed to assess the effect of SiO_2_@CDs on L02 cell proliferation (Fig. 7A). The results indicated that SiO_2_@CDs did not cause a significant decrease in cell viability. For instance, even at high concentrations, such as 100 mg/L and 1,000 mg/L, cell viability remained high, above 90% and 85%, respectively, after 48 h of exposure. Toxicity grading based on the relative growth rate (RGR) values and the United States Pharmacopeia (USP) criteria revealed that SiO_2_@CDs were generally non-toxic (grade “0”), except at the highest concentration of 1,000 mg/L where they exhibited slight cytotoxicity (grade “1”) (**Table S4**). Furthermore, the flow cytometry results in Fig. 7B supported these findings, showing no significant increase in the rates of cell death in the SiO_2_@CDs-treated group compared to the control group. The Calcein-AM and PI dual staining assay visually confirmed cell viability (Fig. 7C) with green fluorescence indicating live cells and red fluorescence indicating dead cells. The majority of cells in both the CK and SiO_2_@CDs-treated groups appeared green, indicating high cell viability. Only a small number of cells in both the SiO_2_@CDs-treated group and the control group exhibited red fluorescence, with no noticeable difference between the two groups, confirming the low cytotoxicity of SiO_2_@CDs.

## Conclusion

In this study, we developed composite nanomaterials (SiO_2_@CDs) and successfully demonstrated their effectiveness in controlling aphid populations and promoting plant growth. Our findings showed that the application of SiO_2_@CDs not only significantly suppressed aphid numbers but also boosted plant defense mechanisms. By incorporating SiO_2_@CDs, we were able to stimulate the physical resistance of plants (Si) and activate internal defense signals, such as phytohormones SA, ultimately leading to improved plant health. Moreover, the use of SiO_2_@CDs triggered oxidative stress in plants by elevating the activity of antioxidant enzymes in response to aphid attack. Through metabolomics analysis, we identified various defense metabolites, including IAA, peroxidase, and beta-galactosidase, which contributed to the enhanced resistance of tobacco plants against aphids. Furthermore, our biosafety assessments demonstrated that SiO_2_@CDs had minimal toxicity towards non-target organisms, such as zebrafish, earthworms, ladybirds, and human cells, highlighting its safety for the environment and human health. These results provide valuable insights into the mechanisms by which SiO_2_@CDs nanocomposites induce plant resistance to aphids, suggesting their potential as a sustainable, eco-friendly pest control solution in agriculture. Despite these promising findings, several aspects require further investigation before field-scale applications can be fully realized. Challenges such as optimizing the large-scale synthesis of SiO_2_@CDs with consistent quality, reducing production costs, and understanding their long-term fate and accumulation in complex agroecosystems need to be systematically addressed. Addressing these issues will be essential for translating the current laboratory-scale success into practical and sustainable solutions for crop protection.

## Electronic supplementary material

Below is the link to the electronic supplementary material.

Supplementary Material 1

## Data Availability

No datasets were generated or analysed during the current study.
