# Supplementary material for: Silicon-coated carbon quantum dots composite nanomaterials mediate pest resistance activation in tobacco (Nicotiana tabacum)
Source: J Nanobiotechnology. 2025 May 19;23:359. doi: 10.1186/s12951-025-03449-0 (PMC12087201; doi:10.1186/s12951-025-03449-0)
Supplement: Supplementary file 1 — Supplementary Material 1 [file 12951_2025_3449_MOESM1_ESM.docx]

Supporting Information

**Silicon-coated Carbon Quantum Dots Composite Nanomaterials Mediate Pest Resistance Activation in Tobacco (*Nicotiana tabacum*)**

Kang-Li He^1,2^, Jing-Han Yang^3^, Fu-Xiao Yu^1,2^, Nuo Wei^4^, Qian-Wei Liang^4^, Jia-Wei Feng^1,2^, Tian-Ci Yi^1^, Xiang-Sheng Chen^1^, Guy Smagghe^1^, Shun-Hua Gui^1,2*^, Tong-Xian Liu^1,2*^

^1^ Guizhou Provincial Key Laboratory for Agricultural Pest Management of the Mountainous Region, Institute of Entomology, College of Agriculture, Guizhou University, Guiyang, 550025, China.

^2^ Institute of Plant Health and Medicine, Guizhou University, Guiyang, China.

^3^ State Key Laboratory of Green Pesticide, Key Laboratory of Green Pesticide and Agricultural Bioengineering, Ministry of Education, Center for Research and Development of Fine Chemicals of Guizhou University, Guiyang, China.

^4^ College of Plant Protection, Yangzhou University, Yangzhou, China.

* Corresponding author E-mail: shgui3@gzu.edu.cn (S.-H. Gui); tx.liu@gzu.edu.cn (T.-X. Liu).

**Table of Contents**

1. Synthesis and Characterization of Nanomaterials (NMs)

2. Effect of NMs on Activity of Antioxidative Enzymes

3. Effect of NMs on Aphids Resistance in Tobacco

4. Parameters of LC-MS for Determining Leaves Metabolites

5. Experimental Data

5.1 Characterization of NMs

5.2 Superior Wettability, Dispersion and Photodegradation Resistance of NMs

5.3 Effect of Supplemental Spraying Treatment on Tobacco Seedlings

5.4 Leaf Metabolic Responses to SiO_2_@CDs Application

5.5 Cytotoxicity Assessment of SiO_2_@CDs

**1. Synthesis and Characterization of Nanomaterials (NMs)**

**Synthesis of Carbon Quantum Dots (CDs)**

CDs were synthesized by dissolving 1.05 g citric acid in 10 mL water, sonicated, then adding 335 μL ethylenediamine and stirring for 10 min. The mixture was heated at 200°C for 10 h, cooled, dialyzed for 3 days using a 500 Da membrane, and freeze-dried.

**Synthesis of Silicon-coated Carbon Quantum Dots (SiO_2_@CDs)**

For the SiO_2_@CDs composite, 100 mg of CDs were dispersed in 125 mL ethanol, sonicated, then mixed with 6.5 mL NH_3_·H_2_O and stirred. After adding 0.25 mL TEOS and stirring for 1 h, a dropwise addition of 4.0 mL TEOS and 10 mL ethanol was made, followed by a 9 h reaction. The product was 8,000 rpm centrifuged, washed, and freeze-dried. SiO_2_ NMs were prepared similarly, omitting the CDs.

**2. Effect of NMs on Activity of Antioxidative Enzymes**

Weigh about 0.1 g of tobacco leaf, add 1mL of extraction solution, homogenize in an ice bath, centrifuge at 8,500 rpm for 10 min at 4℃, take the supernatant and put it on ice for measurement.

**Superoxide Dismutase (SOD)**

The absorbance was measured at 450 nm after spiking according to WST-8 method. Warm up the enzyme counter for more than 30 min and adjust the wavelength to 450 nm. Dilute xanthine oxidase solution with distilled water 1: 49. Preparation of working solution: add 100 μL of WST-8 solution into 20 mL of reagent I (Na_2_HPO_4_·12H_2_O, NaH_2_PO_4_, DTPA solutions) and mix well (the prepared reagent can be stored at 4℃ for one week). Dissolve hypoxanthine solution in 5.0 mL of distilled water. Finally, sample determination (add sample, diluted xanthine oxidase solution, working solution and hypoxanthine solution into 96-well plate), mix thoroughly, and then determine the absorbance value of each tube at 450 nm after standing at room temperature for 30 min.

**Peroxidase (POD)**

Preheat the enzyme marker for more than 30 min, adjust the wavelength to 470 nm, and adjust the zero with distilled water. Preparation of working solution: Mix reagent I (Na_2_HPO_4_·12H_2_O, NaH_2_PO_4_ solutions), reagent II (guaiacol solution) and reagent III (hydrogendioxide solution) according to the ratio of 2.6 mL: 1.5 μL: 1.0 μL before use, preheat at 25℃ for more than 10 min, and use it now. Add 10 μL of sample and 190 μL of working solution into the 96-well plate, mix well, and record the absorbance value A1 at 1 min and A2 at 2 min at 470 nm (the time interval between A2 and A1 is 1 min). Calculate ΔA = A2-A1.

**Catalase (CAT)**

Preheat the instrument for more than 30 min, adjust the wavelength to 240 nm and zero the instrument with distilled water. Before the measurement, the working solution of CAT assay was placed in a water bath at 25℃ for more than 10 min. Prepare a 96-well UV plate, add 10 μL of sample and 190 μL of working solution into the 96-well UV plate, mix well, and record the initial absorbance value A1 at 240 nm and the absorbance value A2 after 1 min. Calculate ΔA = A1-A2.

**Malondialdehyde (MDA)**

Suck 0.3 mL reagent I in 1.5 mL centrifuge tube, then add 0.1 mL sample and mix well. 95°C water bath insulation 30 min (cover tightly, to prevent water loss), placed in ice bath cooling, 10,000 rpm, 25°C, centrifugal 10 min. Absorb 200 μL of the supernatant into a micro quartz cuvette or 96-well plate, and determine the absorbance at 532 nm and 600 nm. It is denoted as A532 and A600, ΔA = A532-A600.

**3. Effect of NMs on Aphids Resistance in Tobacco**

For salicylic acid (SA) analysis, prepare the standard and sample wells. Add 50 μL of standard solution to standard wells, and 10 μL of sample with 40 μL of sample diluent to sample wells. Leave blank wells empty. Add 100 μL of HRP-conjugate reagent to all wells, cover with an adhesive strip, and incubate at 37°C for 60 min. Wash each well five times with 400 μL of wash solution, ensuring complete liquid removal after each wash. After the last wash, blot the plate on clean paper towels. Add 50 μL of chromogen solution A and 50 μL of chromogen solution B to each well, mix gently, and incubate at 37°C for 15 min, protecting from light. Add 50 μL of stop solution to each well. The color should change from blue to yellow. If the color is uneven, gently tap the plate. Measure absorbance at 450 nm within 15 min.

**4. Parameters of LC-MS for Determining Leaves Metabolites**

The LC/MS system for metabolomics analysis is composed of Waters Acquity I-Class PLUS ultra - high performance liquid tandem Waters Xevo G2-XS QTof high resolution mass spectrometer, and the mobile phase A was 0.1% formic acid aqueous solution, the mobile phase B was 0.1% formic acid acetonitrile. The elution gradient was set as follows: 0 min, 95% A; 0.5 min, 95% A; 5.5 min, 50% A; 9 min, 5% A; 10.5 min, 5% A; 12 min, 95% A. The injection volume and the flow rate were 2.0 μL and 400 μL/min. The parameters of the ESI ion source are as follows: Capillary voltage: 2500 V (positive ion mode) or -2000 V (negative ion mode); cone voltage: 30 V; ion source temperature: 100°C; desolvent gas temperature 500°C; backflush gas flow rate: 50 L/h; Desolventizing gas flow rate: 800 L/h.

5. Experimental Data

**5.1 Characterization of NMs**

**Figure S1.** The particle size diagram of CDs.

**Figure S2.** (A) The HRTEM image of the SiO_2_@CDs; (B) The N1s XPS spectrum of the SiO_2_@CDs.

**5.2 Superior Wettability, Dispersion and Photodegradation Resistance of NMs**

**Figure S3.** Zeta potential distribution of SiO_2_.

**Figure S4.** Confocal microscope images of CK (water) and SiO_2_@CD in tobacco leaves.

**5.3 Effect of Supplemental Spraying Treatment on Tobacco Seedlings**

**Table S1.** Summary table of two-way ANOVA results showing the interactive effects of NM treatment and herbivore treatment with aphids on tobacco plant growth, antioxidant enzyme, and phytohormone traits. The test results are shown with the test statistic *F* value and significance levels (****P* < 0.001, ***P* < 0.01, **P* < 0.05 and “NS” indicates no significant difference (*P* > 0.05)).

| Plant traits | NM treatment  df=3 | Herbivore  df=1 | NM treatment × Herbivore  df=3 |
| --- | --- | --- | --- |
| ΔFW | 4.44 ^**^ | 40.10 ^***^ | 0.38^NS^ |
| DW | 2.45^NS^ | 13.75^***^ | 0.48^NS^ |
| Leaf SOD | 2.46^NS^ | 109.90^***^ | 2.48^NS^ |
| Leaf CAT | 0.58^NS^ | 0.66^NS^ | 0.42^NS^ |
| Leaf POD | 3.05^*^ | 16.31^***^ | 1.83^NS^ |
| Leaf MDA | 1.56 ^NS^ | 1.75 ^NS^ | 1.79 ^NS^ |
| Leaf Si | 20.80^***^ | 3.06^NS^ | 0.44^NS^ |
| Leaf SA | 79.92^***^ | 417.59^***^ | 30.31^***^ |

**Figure S5. Effects of different treatments on net photosynthetic rate (Pn) (A) and chlorophyll content (SPAD) (B) in tobacco. Different letters indicate significant differences between treatments (P < 0.05, n = 4).**

**Figure S6**. Feeding bioassay experiment testing the direct entomotoxic effect of NMs at different concentrations (0.1, 1, 10, 100, and 1,000 mg/L) against aphids in leaf-dipping method at (A) 24, (B) 48 and (C) 72 h of treatment (*P* < 0.05, n = 4).

**Figure S7**. Images of control and NM-treated tobacco plants under aphid infestation from day 1 to day 7. Scale bars in all images represent 3 cm.

**5.4 Leaf Metabolic Responses to SiO_2_@CDs Application**

**Figure S8.** KEGG pathway classification chart of major differential metabolites.

**Figure S9.** GO classification statistics column chart. The lower horizontal coordinate in the chart indicates the secondary classification term of the GO or the KEGG pathway. The vertical coordinate indicates the number of genes on that secondary classification by comparison.

**Figure S10.** Bar chart of KEGG enrichment for differentially expressed genes. The KEGG enrichment results of the top 20.

**Figure S11.** Top 30 significantly enriched pathways of differential genes/metabolites – KEGG enrichment bar plot *P* < 0.05.

**5.5 Gene Expression Analysis**

**Table S2.** Expression levels (Count and FPKM) of 7 genes shown in Figure 5C.

| Gene name | SAUR64 | | IAA7 | AUX22 | CSE | CYP84A1 | | At1g64390 | | PME34 | |
| --- | --- | --- | --- | --- | --- | --- | --- | --- | --- | --- | --- |
| CK_Rep1_Count | 333 | 3753 | | 157 | 130 | 35 | | 1097 | | 902 | |
| CK_Rep2_Count | 32 | 2062 | | 40 | 45 | 10 | | 296 | | 566 | |
| CK_Rep3_Count | 69 | 2049 | | 43 | 29 | 9 | | 296 | | 451 | |
| SiO_2_@CDs_  Rep1_Count | 218 | 4040 | | 104 | 105 | 55 | | 763 | | 825 | |
| SiO_2_@CDs_  Rep2_Count | 1188 | 6615 | | 1660 | 388 | 86 | | 2236 | | 2033 | |
| SiO_2_@CDs_  Rep3_Count | 737 | 5034 | | 925 | 203 | 199 | | 1847 | | 1008 | |
| CK_Rep1_FPKM | 13.71379 | 93.36839 | | 3.112619 | 2.367188 | 0.340487 | | 13.40728 | | 11.64442 | |
| CK_Rep2_FPKM | 1.282251 | 59.07259 | | 0.786797 | 0.810197 | 0.290103 | | 3.331246 | | 7.267374 | |
| CK_Rep3_FPKM | 3.063476 | 53.45951 | | 0.919862 | 0.561578 | 0.101436 | | 3.893741 | | 6.261303 | |
| SiO_2_@CDs_  Rep1_FPKM | 37.58312 | 165.6159 | | 22.71947 | 4.560917 | 2.431971 | | 27.11153 | | 16.01834 | |
| SiO_2_@CDs_  Rep2_FPKM | 48.60553 | 174.6098 | | 32.71423 | 7.010388 | 0.837992 | | 27.11295 | | 26.02711 | |
| SiO_2_@CDs_  Rep3_FPKM | 9.983252 | 119.7683 | | 2.291003 | 2.118332 | 0.6029 | | 13.40278 | | 11.86883 | |
| P-value | 0.002102 | 0.001132 | | 0.00000688 | 0.005009 | 0.014582 | | 0.006551 | | 0.000632 | |
| log2FC | 2.4741 | 1.1828 | | 3.6525 | 1.9362 | 2.4713 | 1.7343 | | 1.4631 | |  |

**Table S3.** Expression levels (Count and FPKM) of 7 genes shown in Figure 5C.

| Gene name | PME34 | BGLU12 | PR-3.5 | GPX3 | ADH1 | PER63 | PLAT1 | MO2 |
| --- | --- | --- | --- | --- | --- | --- | --- | --- |
| CK_Rep1_Count | 902 | 592 | 43 | 35 | 1686 | 406 | 1605 | 79 |
| CK_Rep2_Count | 566 | 380 | 24 | 25 | 1136 | 59 | 592 | 8 |
| CK_Rep3_Count | 451 | 503 | 45 | 143 | 1616 | 405 | 419 | 15 |
| SiO_2_@CDs_  Rep1_Count | 825 | 377 | 50 | 112 | 2789 | 161 | 740 | 37 |
| SiO_2_@CDs_  Rep2_Count | 2033 | 500 | 225 | 112 | 7184 | 405 | 3837 | 461 |
| SiO_2_@CDs_  Rep3_Count | 1008 | 377 | 134 | 294 | 1528 | 197 | 2621 | 209 |
| CK_Rep1_FPKM | 11.64442 | 9.309429 | 1.17485 | 3.059428 | 31.81353 | 1.554285 | 21.69387 | 1.362065 |
| CK_Rep2_FPKM | 7.267374 | 6.044113 | 0.164013 | 0.184601 | 20.98174 | 1.489237 | 7.951035 | 0.126825 |
| CK_Rep3_FPKM | 6.261303 | 3.307841 | 0.685169 | 0.755448 | 32.31408 | 2.523171 | 6.098081 | 0.262232 |
| SiO_2_@CDs_  Rep1_FPKM | 16.01834 | 5.096261 | 4.569973 | 6.128566 | 35.00638 | 10.22635 | 43.78357 | 4.471759 |
| SiO_2_@CDs_  Rep2_FPKM | 26.02711 | 5.096261 | 6.175954 | 6.128566 | 132.3175 | 15.43067 | 51.43021 | 7.906453 |
| SiO_2_@CDs_  Rep3_FPKM | 11.86883 | 5.096261 | 1.536435 | 2.676053 | 57.68945 | 6.640971 | 11.14203 | 0.697176 |
| P-value | 0.000632 | 0.005009 | 0.014361 | 0.005571 | 0.002776 | 0.002961 | 0.008565 | 0.002095 |
| log2FC | 1.4631 | 1.2592 | 2.6331 | 2.0675 | 1.4592 | 2.9661 | 1.6484 | 2.9389 |

**5.6 Cytotoxicity Assessment of SiO_2_@CDs**

**Table S4.** Results of cytotoxicity test with human L02 liver cells (n = 3).

| Concentration (mg/L) | OD_450 nm_ | RGR (%) | Toxicity/Grade |
| --- | --- | --- | --- |
| 0 | 2.55 | 99.41 | 0 |
| 0.1 | 2.53 | 99.33 | 0 |
| 0.5 | 2.51 | 98.20 | 0 |
| 1 | 2.45 | 95.67 | 0 |
| 5 | 2.44 | 95.22 | 0 |
| 10 | 2.42 | 94.56 | 0 |
| 50 | 2.42 | 94.30 | 0 |
| 100 | 2.41 | 93.91 | 0 |
| 500 | 2.33 | 90.60 | 0 |
| 1,000 | 2.27 | 87.83 | 1 |

Note:(RGR value ≥ 90%, cytotoxicity grade 0; RGR value of 89% to 75%, cytotoxicity grade 1; RGR value of 50% to 74%, cytotoxicity grade 2; RGR value of ≤ 49%, cytotoxicity grade 3 to 5.)
